# Intraocular Pressure (IOP) in Patients with Acromegaly versus Healthy Controls: A Systematic Review and Meta-Analysis

**DOI:** 10.3390/vision8030054

**Published:** 2024-09-12

**Authors:** Anna M. Kober, Maria Sobol

**Affiliations:** 1 School of Medicine, University of Nottingham, Nottingham NG7 2UH, UK; 2 Department of Biophysics, Physiology and Pathophysiology, Medical University of Warsaw, Chałubińskiego 5, 02-004 Warsaw, Poland

**Keywords:** intraocular pressure, acromegaly, meta-analysis, systematic review

## Abstract

Purpose. Acromegaly is an uncommon condition but affects numerous organ systems. It has been found that patients with acromegaly can experience ocular changes, such as raised intraocular pressure (IOP). Numerous studies have since been carried out to determine whether there is a significant difference between IOP in patients with acromegaly and healthy controls and there is much disagreement in the literature. This study aims to perform a systematic review and meta-analysis to establish whether there is a significant difference in IOP between the two groups in a larger population. Methods. A systematic literature search was performed using PubMed, Scopus, and Web of Science to access relevant databases and to locate outcome studies. Eligibility criteria included type of publication, participant characteristics, and report of outcomes. Data analysis was conducted with a fixed-effects model. Results. Three articles were included in the final analysis. The mean value of IOP corrected for central corneal thickness (IOPcc) for the group of 102 patients with acromegaly was 15.33 with confidence levels of 13.05–17.62 [mmHg]. The mean difference between the control and acromegaly group was 1.17 with confidence levels of 0.64 to 1.70 [mmHg], which was found to be statistically significant (*p* < 0.001). Conclusion. The results of the meta-analysis indicate that acromegaly is associated with increased IOP. As raised IOP is a risk factor for the development of glaucoma, detailed IOPcc evaluation should be an important procedure in the follow-up visits of patients with acromegaly.

## 1. Introduction

Acromegaly is a condition resulting from long-standing exposure to an excess of growth hormone (GH) in the body [1]. In 2021, a systematic review and meta-analysis estimated the global prevalence of acromegaly to be 5.9 per 100,000 persons [2].

The underlying pathology of acromegaly is based on the stimulation of GH-receptors leading to the production of insulin-like growth factor-1 (IGF-1) [1]. In turn, IGF-1 promotes cell proliferation whilst inhibiting apoptosis and this process is responsible for the majority of the main clinical manifestations [1]. Although gigantism follows the same disease process, it is defined by starting in childhood [3]. The most common cause of acromegaly is a GH-secreting pituitary tumour, of which there are many variants [1]. Other pituitary-related causes of acromegaly are pituitary hyperplasia and neoplasia, and although most cases are sporadic, they may be predisposed in some familial syndromes [1]. Moreover, another cause of excess GH leading to acromegaly may be due to overstimulation of the pituitary gland by GH-releasing hormone, although this is relatively uncommon in clinical practice [1]. However, some conditions associated with this disruption of the hypothalamic-pituitary axis are hypothalamic gangliocytomas as well as neuroendocrine tumours of the lung, pancreas and thyroid and pheochromocytomas [1]. In addition, ectopic production of GH is rare but has been previously noted in cases of lymphoma and neuroendocrine pancreatic tumours [1]. 

The insidious onset and slow progression of the disease means that it is often diagnosed four to ten years after the initial beginning of the disease process [3]. Measurement of serum IGF-1 and finding raised serum GH after an oral glucose tolerance test allow a diagnosis to be made [3,4,5]. Appearance-related features suggestive of acromegaly that may be noticed by the patient or friends and family include broadening of the hands and feet, thickening of skin, and facial changes such as widened nose, prominent cheekbones, frontal bossing, thicker lips, and marked facial lines [3]. Healthcare professionals also have a key role in identifying acromegalic changes in patients. For example, dentists may recognise features such as mandibular prognathism, jaw malocclusion, maxillary widening, and tooth separation [3,6]. Similarly, ophthalmology is another speciality which can have an important role in assessing for acromegaly. The literature has noted numerous ocular features occurring directly from the acromegalic pathophysiology, local effects such as optic chiasm compression, and associated co-morbidities such as diabetes [7,8,9,10,11,12,13,14]. Ocular changes include raised intraocular pressure (IOP), increased central corneal thickness (CCT), diabetic retinopathy, pigmentary degeneration of the retina, choroidal thickening, choroid melanocytic tumours, bilateral hemianopia, ptosis, restrictive extraocular myopathy with diplopia, enlargement of extraocular muscles, and ptosis [7,8,9,10,12,14,15,16,17,18,19,20,21,22,23,24]. 

The first reported case of raised IOP associated with acromegaly was in 1955 [19]. Since then, numerous studies have aimed to determine whether there is a significant difference between IOP in patients with acromegaly and healthy controls and there is disagreement in the literature [7,8,10,12,16,17,24,25,26]. IOP can be easily assessed in clinical practice, and it has been suggested that all adults attending an eye unit should have IOP measured unless there are any contraindications, such as a corneal ulcer or trauma [27]. The current gold standard for measuring IOP is Goldmann applanation tonometry; however, other methods have also been described [28]. 

Raised IOP is a major risk factor for the development of glaucoma, which is one of the leading causes of visual impairment and blindness worldwide [29]. Since there is disagreement between individual studies, this systematic review and meta-analysis aims to establish in a larger group of patients whether there is a significant difference between IOP measurements in patients with acromegaly and healthy controls. 

## 2. Materials and Methods

**Figure 1:**
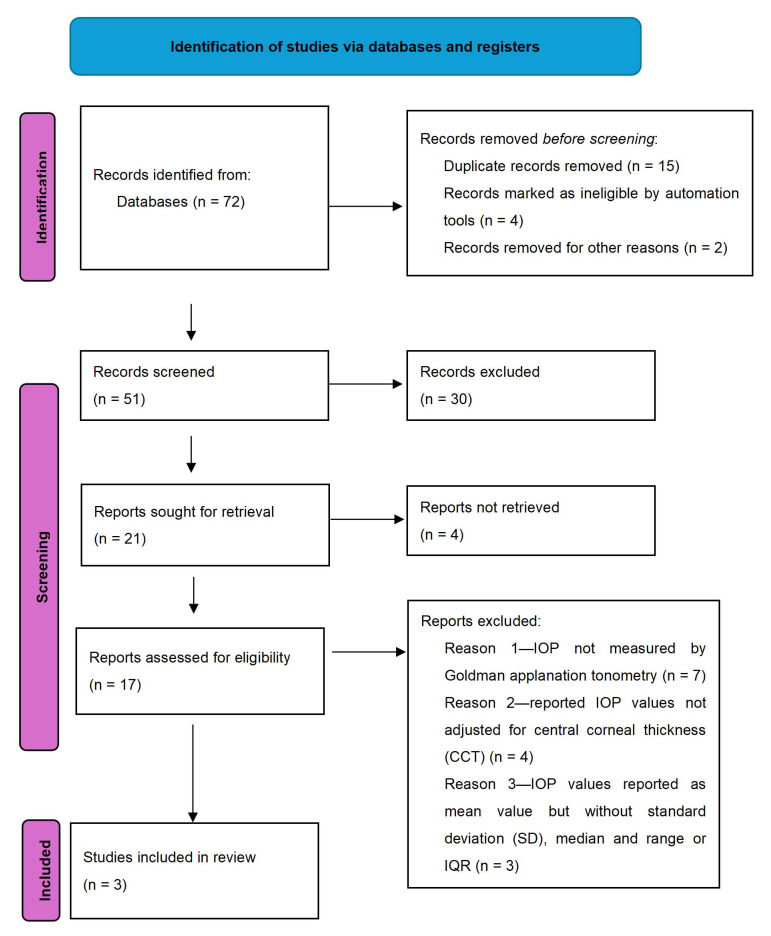
Flow chart for inclusion of articles.

Studies included in this research were selected from a systematic search of literature in Scopus, Web of Science, and PubMed. Studies published up until 11 June 2023 were considered. The inclusion criteria were as follows: English language, original papers, human studies, retrospective papers, cross-sectional and case-control studies. The screening of the results was based on the words/phrases ‘acromegaly’ and ‘intraocular pressure (IOP)’. Studies were excluded if the IOP was not measured by Goldman applanation tonometry and the reported IOP values were not adjusted for central corneal thickness (CCT) [30]. For the analysis, studies which only reported the results as median and range, median and interquartile range (IQR) of IOP corrected for CCT (IOPcc), or mean value but without standard deviation (SD) were not included (Figure 1). Unpublished reports, abstracts, and case reports were not considered. Authors were not contacted. Two reviewers, the first and the second author of this article, assessed each abstract and full text for potential inclusion and reached a consensus for the articles to be included in the final review.

### 2.1. Study Selection

The systematic review was conducted using the PRISMA guidelines [31]. Specific requirements are listed below: 

Study group: subjects with a confirmed clinical diagnosis of acromegaly.

Control group: age-matched healthy subjects with no history of ocular surgery or refractive error > 3.00 dioptres. 

Limits used: Human subject studies published in English.

Timing: studies published up to and inclusive of 11 June 2023.

### 2.2. Statistical Analysis

Statistical analysis was performed using the Statistica 13 package Dell software. The Q test was used to test heterogeneity, and I^2^ statistics were calculated to quantify and evaluate the heterogeneity (low: 25–50%, moderate: 50–75%, and high: >75%). Since heterogeneity exceeded 0%, the analysis was conducted with a fixed-effects model and the standardised mean and mean differences were given with 95% confidence intervals (95% CI). Forest plots were generated to showcase the differences between the acromegaly and control groups for the IOP_CCT_ parameter and corresponding 95% confidence intervals (CIs) for each study as well as overall estimates. To assess the stability of the plotted results, sensitivity analysis was conducted by excluding each study at a time. To assess for publication bias, Egger’s and Begg’s tests were conducted.

## 3. Results

The search strategy identified 72 articles among the PubMed, Scopus, and Web of Science databases. After screening using the phrases ‘acromegaly’, ‘mean value and SD IOPcc’, and ‘Goldman applanation tonometry’, three studies were selected and hence included [12,24,32].

Table 1 summarises the characteristics of the included studies. The study groups included patients with acromegaly whilst only healthy patients were included in the control groups. All participants in the control groups were age-matched with the subjects. Patients who had refractive errors of more than three dioptres, had systemic or ocular disease (including surgery or history of ocular trauma), or used ophthalmic or systemic drugs were excluded from the studies. The participants in the control groups were recruited from patients who were presenting for routine eye examinations [12,32] or simple ocular complaints such as presbyopia or dry eyes [24] or from the hospital staff and their families [17]. All of the studies included in the analysis were from Turkey.

In the meta-analysis conducted for patients with acromegaly, 102 patients were included. The age range of the patients was 22 to 69 years. The study sample sizes varied from 31 to 36 patients.

Two authors, Yazgan et al. [24] and Kilic et al. [32], provided the results for the right eye only while Sen et al. [12] did not specify which eye measurements were used to report IOPcc. 

**Figure 2:**
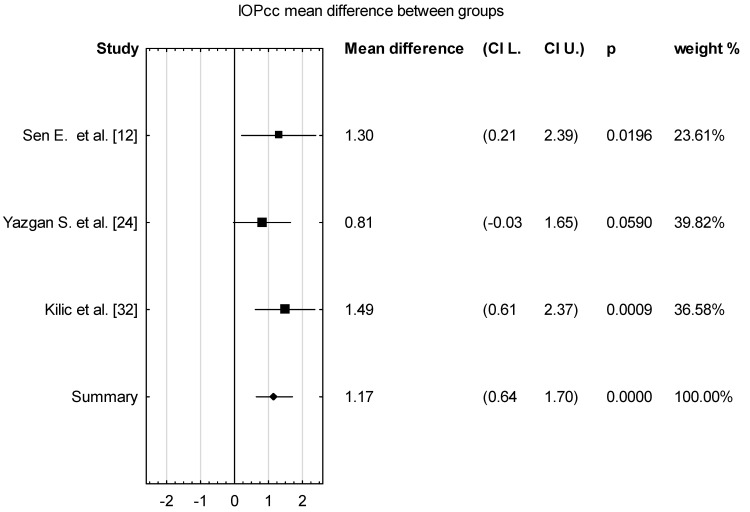
Forest plot of IOPcc for patients with acromegaly, *p* value indicating level of statistical significance. The size of the box represents the point estimate for each study in the forest plot and is proportional to that study’s weight-estimate contribution to the summary estimate. Horizontal lines represent 95% CI.

**Figure 3:**
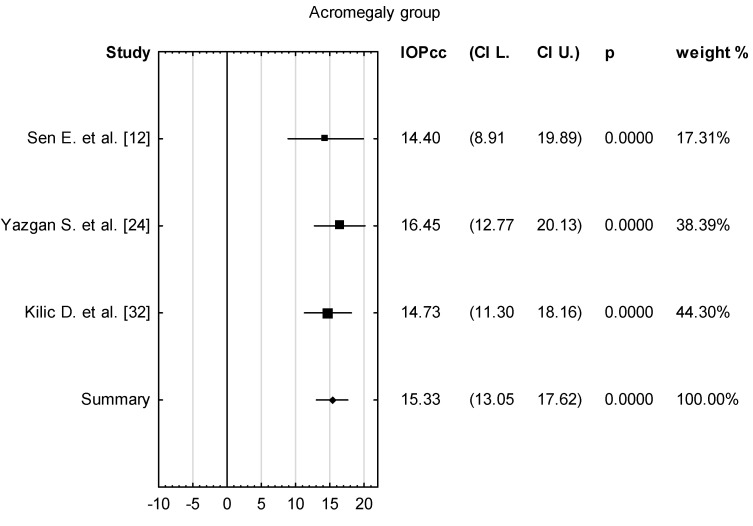
Forest plot of mean difference of IOPcc between acromegaly and control group, *p* value indicating level of statistical significance. The size of the box represents the point estimate for each study in the forest plot and is proportional to that study’s weight-estimate contribution to the summary estimate. Horizontal lines represent 95% CI.

The mean value of IOPcc for the group of 102 patients with acromegaly was 15.33 with confidence levels of 13.05–17.62 [mmHg] (Figure 2). The mean difference for the control and acromegaly groups was 1.17 with confidence levels of 0.64 to 1.70 [mmHg] (Figure 3), which was found to be statistically significant (*p* < 0.001)

### Risk-of-Bias Assessment

**Figure 4:**
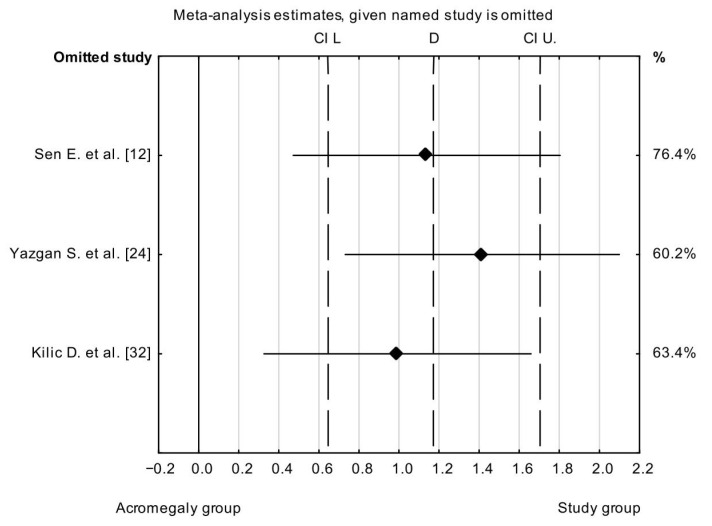
Sensitivity analysis for the effect of individual studies on the pooled difference of IOP_CCT_ with confidence intervals. The named study on the Y-axis is omitted from the analysis to assess the effect it has on the overall results.

The result of the sensitivity analysis showed that the IOP_CCT_ mean difference between the acromegaly and control groups varied from 1.0 mmHg (95% CI: 0.33 to 1.66 mmHg) to 2.0 mmHg (95% CI: 0.73 to 2.10 mmHg), when Kilic et al. [32] and Yazgan et al. [24] were excluded, respectively. This indicates that the stability of the mean difference between the acromegaly and control groups was not influenced by a single study (Figure 4). An Egger’s publication bias was generated, and the visual symmetry of the funnel plot suggested that there was minimal publication bias. The results of Egger’s test (*p* = 0.752) and Begg’s test (*p* = 0.602) also indicated that there was minimal potential risk of publication bias.

## 4. Discussion

Ocular involvement in patients with acromegaly, in particular raised IOP, has been known for more than 65 years. Since Arén et al.’s [19] first report in 1955 suggesting that there is a significant difference in IOP measurement between patients with acromegaly and healthy controls, numerous studies have also looked at the relationship and there is much disagreement in the literature. In their individual studies, Sen et al. [12] and Kilic et al. [32] reported a significance difference in the IOPcc values between the study and control groups, but Yazgan et al. [24] found no significant difference between the two groups. Furthermore, as acromegaly is a rare condition, the group sizes in each study were relatively small. Only one study stated the sample size required to achieve a power of 80% [12]. Since raised IOP is a risk factor for significant complications such as glaucoma, a systematic review and meta-analysis was carried out to establish on a larger population of patients whether there was a significant difference in IOPcc values between patients with acromegaly and healthy controls and therefore whether patients with acromegaly may be at an increased risk of further ocular complications.

It is well known that IOP measurements can be affected by CCT [33]. In addition, acromegaly itself may change the anatomy and physiology of eye tissue, meaning it can impact corneal characteristics, which can affect IOP readings. As a result, it was essential for the authors to report IOPcc, meaning IOP values adjusted for CCT. Sen et al. [12] stated that the Dresdner correction formula [30] was used to calculate IOPcc values from IOP and CCT readings, Yazgan et al. [24] used the Doughty and Zaman formula [34] and Kilic et al. [32] reported IOPcc as calculated by machine software which was based on the corrections suggested by Ehlers et al. [33]. Although according to the European Glaucoma Society 5th Edition Guidelines [35] published in 2023, IOP correction algorithms based on CCT which are not validated should be avoided, it is important to note that CCT continues to be an easily accessible parameter to measure which can guide clinicians to obtain relatively accurate IOP outcomes when the hardware environment is limited.

Regarding recruitment of participants, the studies did not specify their selection methods other than the population source and exclusion criteria. As a result, it is unclear whether there is any selection bias.

The reported values of IOP in patients with acromegaly varied between the different studies [12,24,32]. This could be because each study considered patients with different lengths of disease duration. The mean duration of disease was 4.3 ± 2.4 years in the study of Sen et al. [12], 9.8 ± 3.6 years in Yazgan et al.’s [24] study, and 9.50 ± 3.90 years for Kilic et al. [32]. The association between IOP changes and disease duration has been investigated in various studies. In their research, neither Polat et al. [16] nor Altinkaynak et al. [17] found a significant correlation between disease duration and IOP readings which had been corrected for CCT. Furthermore, Ozok et al. [36] considered subjects with controlled and uncontrolled acromegaly but there was no statistically significant difference in IOPcc values between the two groups. In addition, Polat et al. [16] and Altinkaynak et al. [17] analysed patients with active and inactive disease status. Although both authors measured greater IOPcc values in the active acromegaly group compared with the inactive disease patients, the difference was not statistically significant in either study [16,17]. In all of the studies considered for the meta-analysis, the control group subjects were age- matched to the acromegaly patients and there was no statistically significant difference between the two groups [12,24,32]. All of the studies also ensured that the control group was gender-matched to the subjects and they found no statistically significant difference between the groups in this regard [12,24,32].

Regarding selection for the meta-analysis, the studies of Pekel et al. [8] and Akay et al. [26] were not considered, as the methodology was unclear and therefore it was uncertain whether the IOP values were corrected for CCT. Additionally, Altinkaynak et al. [17], Erol et al. [18], Kan et al. [25], and Ozok et al. [36] did not report IOP values corrected for CCT. Furthermore, Quaranta et al. [10] was not included, as the IOPcc values were provided as median (range), nor was Polat et al. [16], which reported its results as median (minimum – maximum), or Ciresi et al. [7], which provided data as median (IQR).

There are limitations of this study. In particular, all of the studies were based in Turkey, meaning that the results may potentially not be applicable to a wider population who have different characteristics. In addition, none of the studies detailed their process for participant recruitment, therefore it is uncertain whether the groups were representative of the respective source populations. However, the studies did report no significant differences between the study and control groups in the baseline characteristics, such as age and gender [12,24,32]. Although some selection criteria were similar across the studies, the control groups were not homogenous, as different sets of criteria were used in each study. Some authors also had very specific exclusion criteria regarding ocular parameters [24,32]. Moreover, the authors also had different methods of correcting IOP for CCT and this may have also affected their results. Finally, this meta-analysis only considered IOP outcomes, without taking into consideration other glaucomatous signs, such as retinal nerve fibre layer (RNFL) or visual field defects. As such, this may somewhat limit the clinical value of the study if it is taken as stand-alone.

The results of this study show that there is a significant difference in IOPcc between patients with acromegaly and healthy controls. This may suggest that patients with acromegaly can be at an increased risk of developing further ocular complications such as glaucoma and hence may need closer monitoring. Furthermore, raised IOP could be a marker to suggest acromegaly in undiagnosed patients. Similarly, in addition to family friends who notice appearance changes and dentists who identify dental changes, opticians could now play a role in recognising ocular changes and considering acromegaly as a differential diagnosis, in particular perhaps in the context of other distinguishing features or in the absence of other clear causes. Future research could also investigate whether there is a significant difference in IOPcc between patients with active and inactive disease states to determine whether this change can be reversible.

## 5. Conclusions

In summary, this study indicates that the patients with acromegaly had a statistically significant higher IOPcc value than the healthy controls. The mean difference between the control and acromegaly groups was found to be 1.17 with confidence levels from 0.64 to 1.70 [mmHg] (*p* < 0.001).

## Figures and Tables

**Table 1:** Summary of the characteristics of the acromegaly and control groups.

	Acromegaly Group			Control Group			Duration of Disease (Mean ± SD) [years]
Study	IOP (Mean ± SD) [mmHg]	Number of Subjects	Age (Mean ± SD) [years]	IOP (Mean ± SD) [mmHg]	Number of Subjects	Age (Mean ± SD) [years]	
Sen et al., 2014 [12]	14.4 ± 2.8	35 (F = 18, M = 17)	42.8 ± 11.9	13.1 ± 1.8	36 (F = 16, M = 20)	38.1 ± 8.1	4.3 ± 2.4
Yazgan et al., 2018 [24]	16.45 ± 1.88	31 (F = 13, M = 18)	41.32 ± 7.22	15.64 ± 1.51	32 (F = 19, M = 13)	41.06 ± 6.15	9.8 ± 3.6
Kilic et al., 2021 [32]	14.73 ± 1.75	36 (F = 28, M = 8)	44.03 ± 9.35	13.24 ± 2.11	40 (F = 31, M = 9)	43.38 ± 8.45	9.5 ± 3.9

## Data Availability

All data generated or analysed during this study are included in this published article.
